# The incidence of anaerobic bacteria in adult patients with chronic sinusitis: A prospective, single-centre microbiological study

**DOI:** 10.1556/1886.2020.00010

**Published:** 2020-06-05

**Authors:** EDIT URBÁN, MÁRIÓ GAJDÁCS, ATTILA TORKOS

**Affiliations:** 1 Department of Public Health, Faculty of Medicine, University of Szeged, 6720 Szeged, Dóm tér 10., Szeged, Hungary; 2 Institute for Translational Medicine, Medical School, University of Pécs, 7624 Pécs, Szigeti ut 12., Pécs, Hungary; 3 Department of Pharmacodynamics and Biopharmacy, Faculty of Pharmacy, University of Szeged, 6720 Szeged, Eötvös utca 6., Szeged, Hungary; 4 Department of Oto-Rhino-Laryngology and Head-, Neck Surgery, Faculty of Medicine, University of Szeged, Szeged, Hungary

**Keywords:** anaerobic bacteria, chronic sinusitis, prospective, microbiology, *Prevotella*, Porphyromonas, otolaryngology

## Abstract

**Introduction:**

Chronic sinusitis caused by anaerobes is a particular concern clinically, because many of the complications are associated with infections caused by these organisms. The aim of this study was to evaluate the incidence of anaerobic bacteria in chronic sinusitis in adults as a part of a prospective microbiological study.

**Materials and methods:**

Over a one-year period, aspirations of maxillary sinus secretions and/or ethmoid cavities were derived in *n =* 79 adult patients with chronic sinusitis by endoscopy in a tertiary-care teaching hospital in Hungary. The qualitative and quantitative compositions of the total cultivable aerobic and anaerobic bacterial and fungal flora cultured on the samples were compared. Correct anaerobic species level identifications were carried out according to standard methods.

**Results:**

Bacteria were recovered for all of the 79 aspirates and the numbers of the significant cultured isolates (with colony forming units ≥10^3^) were between 1 and 10. A total of 206 isolates, 106 anaerobic and 100 aerobic or facultative-anaerobic strains were isolated. The most common aerobic bacteria were *Streptococcus pneumoniae (n =* 40), *Haemophilus influenzae (n =* 29), *Moraxella catarrhalis (n =* 6), *Staphylococcus aureus (n =* 7) and *Streptococcus pyogenes (n =* 6). The anaerobic bacteria included black-pigmented *Prevotella* spp. and *Porphyromonas* spp. (n = 27), *Actinomyces* spp. (n = 13), Gram-positive anaerobic cocci (n = 16), *Fusobacterium* spp. (n = 19) and *Cutibacterium acnes (n =* 8).

**Conclusions:**

This study illustrates the microbial dynamics in which anaerobic and aerobic bacteria prevail and highlights the importance of obtaining cultures from patients with chronic sinusitis for guidance in selection of proper antimicrobial therapy.

## INTRODUCTION

Chronic sinusitis (CS) is an inflammatory disorder of the upper airways, which lasts longer than 12 weeks, often causing residual damage to the sinus mucosa, leading to long-term symptoms (according the definition of the International Rhinosinusitis Advisory Board) [1, 2]. Based on literature findings, chronic sinusitis is almost always accompanied by concurrent nasal airway inflammation, and is often preceded by symptoms of rhinitis; thus, the term chronic rhinosinusitis (CRS) has evolved to more accurately describe this condition [1]. CRS is a multifactorial morbidity, in which the complex microbiome plays a pathogenic role [2]. It is a frequent bacterial infection among adults, affecting approximately 5% of the Western population; the overall prevalence of CRS in the United States is 146/1,000 population [3]. This involves nearly 30 million US adults annually, accounting for approximately 20 million office visits and 1.2 million hospital visits, making CRS more common than any other chronic condition, and for unknown reasons, the incidence of this disease appears to be increasing. According to the data from the US, the ratio of the recurrence is around 25% and the ratio of therapy-resistant cases of CRS is 10–15%. The European Position Paper on Rhinosinusitis and Nasal Polyps 2007 (EPOS 2007) found that the prevalence of CRS to be around 15–16% (this is in part, mostly speculative because of the non-uniformity in symptoms criteria and definitions), among which, diagnosis by general practitioners was only around 2–4% [4].The European prevalence by the EPOS and/GA(2)LEN epidemiological study criteria was estimated to be 10.9% overall and ranging between 5 and 15% in different countries [4, 5]. Based on the results of the National Ambulatory Medical Care Survey of the Centers for Disease Control and Prevention (CDC), rhinosinusitis is the fifth most common cause for the prescription of antibiotics [6]. CRS begins with an inflammation of the mucous membranes in the sinuses, the air-filled passages around the nose and throat, leading to mucous stagnation in the sinus, which forms a rich medium for the growth of various pathogens [1–3]. This early stage of sinusitis is often caused by a viral infection, generally lasting up to 10 days, completely resolving in 99% of cases; however, a small number of patients may develop a secondary acute bacterial infection, which is generally caused by aerobic bacteria [1, 2]. The inflammation causes fluid build-up, eventually plugging the sinus cavity and preventing normal mucus drainage. CRS may be caused by infections of the upper respiratory tract — the nose, pharynx, sinuses and throat — but there are some non-infectious triggers, such as allergens, toxins and underlying genetic predisposition. Approximately 10% of all sinusitis cases are the result of an odontogenic process, with several reports in the literature stating that up to 40% of all sinusitis cases may have an underlying dental pathology [7, 8]. The pathophysiology of this condition is still poorly understood, with multiple environmental, host and microbial factors being implicated: allergies are a common cause, and anatomical problems such as a deviated nasal septum can bring on chronic sinusitis, other suspected causes putative pathological factors include changes in the microbiota, imbalance of the local or systemic immune system, and the presence of moulds or other fungi in the sinuses [9]. There are a lot of the different studies clarifying some microbiological aspects of acute and chronic sinusitis, including its pathophysiology, epidemiology, role of bacterial biofilms and more recently, the microbiome of healthy and/or diseased sinuses. The dysbiosis of intramucosal microbiomes, the presence of biofilms and super-antigens have all been suggested to play a main role in the pathogenesis of CRS: while quantitatively, there are no relevant differences, there were qualitative differences observed in the composition of the sinus microbiota among healthy and CRS-patients [9]. Defining the nature of the role of the microbiota in CRS is important because of the associated therapeutic implications. *Streptococcus pneumoniae, Haemophilus influenzae, Moraxella catarrhalis*, Corynebacterium spp., *Staphylococcus* epidermidis and members of the Enterobacterales order have been noted as the predominant aerobic pathogens recovered from patients with sinusitis; however, with the exception of *Staphylococcus* aureus, the association between any single species and CRS is tenuous [10]. Many of these bacteria can interfere with the overgrowth of potential other pathogens and may play a role in preventing the development of infections. Most cases of CRS are due to acute sinusitis that either is untreated or does not respond to treatment [11]. However, when sinusitis becomes chronic, these organisms are replaced by a variety of both aerobic and anaerobic bacteria and it has been suggested that anaerobic bacteria play a significant role in the pathogenesis of CRS [12]. This may be the result of the selective pressure of antimicrobial agents, sometimes redundantly used in the management of acute viral sinusitis, that enables resistant anaerobic organisms to survive, and over time, for the development of conditions appropriate for anaerobic growth, which include the reduction in oxygen tension and an increase in acidity within the sinus cavity [11, 12]. CRS caused by anaerobic bacteria is a particular concern clinically because many of very serious complications associated with this condition (spread of infection into the bones of the face, mucocele formation, osteomyelitis, meningitis and/or and brain abscess) are associated with these microorganisms [8]. Because of the special techniques required for the collection, transport and culture of anaerobes, the availability of reliable data on anaerobic bacteria associated with CRS, especially in adult patients is limited; however, based on various reports, anaerobic pathogens were recovered in 8–93% of cases [12–14]. The variability in their recovery rate may be due to differences in the methodologies used for sample preparation, transportation, laboratory possibilities of culturing and identification, patient population, different geography and previous surgical and/or antimicrobial therapy.

The evaluation of the pathogenic role of anaerobic bacteria in the acute exacerbation of CRS is of utmost importance. Establishing the correct microbiological diagnosis of sinusitis is of primary importance, as it can serve as a guide to the choice of adequate antimicrobial therapy. Therefore, the aim of our study was to assess the microbial aetiology of CRS in at a tertiary-care hospital in Hungary over a one-year long period.

## MATERIALS AND METHODS

### Study design, details of the clinical centre

A prospective study was undertaken to evaluate the pathogenic role of anaerobic bacteria in the acute exacerbation of CRS in our local settings. The Institute of Clinical Microbiology was the National Reference Laboratory of Human Pathogenic Anaerobic Bacteria in Hungary during the study period. The Institute is a routine diagnostic microbiological laboratory, servicing a 1,820-bed tertiary-care university-teaching hospital in Szeged, Hungary. This Clinical Centre is responsible for the medical care of about 600,000 patients in the southeast region of Hungary (urban and rural population: around 1.3 million people based on the most recent census data).

### Patients, exclusion criteria

During a one-year period, 79 adult patients (45 males, 34 females), ranging in age from 18 to 84 years (mean age: 28.6 years) with CRS (patients corresponding to the following criteria: typical clinical symptoms of sinusitis, i.e. fever, headache, nasal drainage, positive radiographic findings, maxillary sinus and biopsy specimens demonstrating chronic inflammation of the sinus mucosal lining, or clinical and radiologic findings compatible with maxillary sinusitis followed by clinical and radiologic improvement following surgery) were included in the study. Sinusitis was considered chronic if symptoms persisted for ≥12 weeks. Patients were excluded from the study if they were immunocompromised, if the previous or current use of antibiotics was known or if the presence of nasal polyps was known.

### Cultivation and identification of bacterial isolates

Aspirate samples were obtained by the aspirations of maxillary sinus secretions and/or ethmoid cavities by endoscopy. Specimens were aspirated by use of a syringe, with instillation of non-bacteriostatic saline, if necessary. Sinus aspirate samples were injected into reduced transport medium (Portagerm Multitransport Medium/bioMérieux, Marcy l’Etoile, France) and sent to the microbiology laboratory immediately after collection. All samples were processed within 1 h of sampling. Samples were suspended in 1 mL of reduced BHI broth (Brain Heart Infusion broth, with a pH adjusted to 7.2; Oxoid, Basingstoke, United Kingdom) and after gentle dispersion these suspensions were diluted (10^–1^–10^–6^) in pre-reduced BHI broth [8]. The 100 μL of each dilution and 100 μL of the corresponding undiluted suspension were plated immediately on selective and non-selective media. Columbia agar base (Oxoid, Basingstoke, UK) supplemented with 5% (v/v) cattle blood was used to isolate the total cultivable facultative and aerobic bacterial flora. Samples were also plated on Schaedler agar (bioMérieux, Marcy l’Etoile, France) containing horse blood 5% v/v, haemin and vitamin K_1._ For the isolation of anaerobic organisms, these cultures were set up and incubated in an atmosphere of 90% N_2_, 5% H_2_ and 5% CO_2_ in an anaerobic environment (Concept 400 anaerobic incubator, Biotrace International Plc., UK) for 5–7 days at 37 °C. For the selective growth of aerobic Gram-positive cocci and Enterobacterales, blood agar (Oxoid, Basingstoke, UK) and for the selective growing of Enterobacterales, eosin methylene-blue agar (EMB; bioMérieux, Marcy l’Etoile, France) were applied, respectively. Fungal isolates were selectively cultured on Sabouroud Dextrose agar (SDA, bioMérieux, Marcy l’Etoile, France).

For aerobic bacteria, the plates were cultured at 37 °C in a 5%CO_2_-containing environment for 48 h. The selective agar media for the isolation of Enterobacterales were incubated at 37 °C for 24 h. SDA plates were incubated at 37 °C in ambient air for 24 h and additionally, at room temperature for a further 5 days. The results from Gram-staining and the atmospheric growth requirements of each colony type were used to determine the additional biochemical tests required to identify the isolates. API 20A, ATB ID 32 ANA (bioMérieux, Marcy l’Etoile, France) tests were used to identify anaerobic bacteria, facultative anaerobic Gram-positive cocci and bacilli. The VITEK 2 Compact ID/AST (bioMérieux, Marcy l’Etoile, France) automated system was used to identify aerobic bacteria and fungi. Identification of anaerobes was performed based on the Wadsworth-KTL Anaerobic Bacteriology Manual, in addition to matrix-assisted laser desorption/ionisation time-of-flight mass spectrometry (MALDI-TOF MS) [8, 15, 16]. The methodology of sample preparation for mass spectrometry measurements was described elsewhere [8, 15]. Mass spectrometry was performed by the Microflex MALDI Biotyper (Bruker Daltonics Gmbh., Bremen, Germany) in positive linear mode across the *m/z* range of 2–20 kDa; for each spectrum, 240 laser shots at 60 Hz in groups of 40 shots per sampling area were collected. The MALDI Biotyper RTC 3.1 software (Bruker Daltonics Gmbh., Bremen, Germany) and the MALDI Biotyper Library 3.1 were used during spectrum analysis. We regarded the isolated bacterial strains as significant pathogens, if the bacterial colony count was higher than 10^3^ colony forming units (CFU)/mL [8, 15, 16].

### Ethical considerations

As a part of this study, data on the affected patients were also collected, which was limited to their demographic characteristics only (age, sex). The study was deemed exempt from ethics review by the Institutional review board and informed consent was not required as data anonymity was maintained.

## RESULTS

Significant number of cultivable bacteria and/or fungi were recovered from all of the *n* = 79 clinical samples received during the study period. Aerobic or facultative anaerobic bacteria were cultured from *n* = 41 samples (51.9%), aerobic and anaerobic mixed flora was cultured in *n =* 36 cases (45.6%) and only two patients had anaerobic bacterial flora exclusively (2.5%). A total of 106 anaerobic strains and 100 aerobic-, or facultative anaerobic bacterial strains were isolated. The average number of organisms isolated per patient was 2.61 and the number of cultured isolates varied between 1 and 10; the 106 anaerobic strains that belonged to 29 different species were cultured from 36 patients. Only one bacterial strain was isolated in significant colony counts from each of 32 patients (49.4%), 30 of these pathogens were aerobes and only 2 were anaerobes.

The most common isolated aerobic bacteria were *S. pneumoniae (n =* 40; 50.6%), *H. influenzae (n =* 29; 36.7%) and *M. catarrhalis (n =* 6; 7.6%); *S. aureus (n =* 7; 8.7%) and *Streptococcus pyogenes (n =* 6; 7.6%) strains were also isolated in lower numbers. Some Gram-negative enteric rods were also found in this study, including *Klebsiella pneumoniae, Serratia marcescens, Escherichia coli* and *Citrobacter* spp. *(n =* 9; 11.4% altogether). Because these organisms are rarely found in sinus cultures originating from normal individuals, their isolation from these symptomatic patients suggests a potential pathogenic role. Only a few of the patients had significant colony counts for pathogenic yeasts: *n =* 1 *Candida albicans* and *n =* 2 *Candida glabrata* strains were isolated from three different patient’s samples.

The predominant anaerobic isolates were pigmented *Prevotella* and *Porphyromonas* spp. *(n =* 27 altogether), *Fusobacterium* spp. (n = 19), especially *Fusobacterium nucleatum (n =* 12) and numerous Gram-positive anaerobic cocci (GPAC) *(n =* 16) (Table 1). The most common anaerobic microorganisms isolated from these samples accounted for 58.8% all of the anaerobic strains in this study. Unusually high number of *Actinomyces* spp. strains *(n =* 13) were also isolated (12.3%); interestingly one of them was the single cause of the syndrome in a very high colony forming unit count (10^6^ CFU/mL). Typical anaerobic odontopathogenic strains (e.g. *Veillonella parvula, Leptotrichia buccalis, Eikenella corrodens* and *Eggethella lenta)* were isolated in the same numbers *(n =* 2; 2.6%, respectively). Only four isolates belonged to the genus *Bacteroides: n =* 2 of them were *Bacteroides fragilis* and *n =* 2 were *Bacteroides ureolyticus.* Surprisingly, *n =* 3 clostridial strains were also isolated: *n =* 1 *Clostridium sordelli* and *n =* 2 *Clostridiun butyricum* isolates, which are not common in this infection, according to the recently published data.

## DISCUSSION

In contrast to the well-established roles of microbes in the aetiology of acute sinusitis, the exact roles of the abovementioned microorganisms (namely *Prevotella* and *Porphyromonas* spp., *Fusobacterium* spp., GPAC, *V. parvula, L. buccalis, E. corrodens, E. lenta, Bacteroides* spp. and *Clostridium* spp.) in the aetiology of CRS are uncertain [1, 2]. Various researchers disagree on the microbial aetiology of CRS; some of the disagreement may be explained by the different methodological approaches to the processing of the obtained microbiological samples. Many bacterial organisms have been identified in the sinus tracts of patients with CRS and are reported in the literature, but there is no consensus as to their correct pathogenic role. Despite the exact cause of the inflammation associated with CRS is uncertain, the presence of bacteria within the sinuses has been well documented in different studies [9, 10]. Some of these studies have examined the bacterial pathogens associated with CRS, but most of these reports did not employ methods for isolation adequate for the recovery of strict anaerobic bacteria. Studies that have used adequate methods for isolation of anaerobes have demonstrated their prominence in CRS, while those that did not use such methods have failed to recover them. Immunosuppressed patients have episodes of sinusitis caused by the usual agents associated with acute sinusitis in immunocompetent patients and they may also become infected with a broad array of unusual microorganisms, including mycobacterial species, fungi and sometimes protozoa. According to certain data from the literature, the presence of anaerobic bacteria in CRS in adults is often clinically significant [11]. Initial studies by Frederick and Braude in the 1970s implicated polymicrobial bacterial flora and emphasised the pathogenic importance of different anaerobic species in particular [13].

Previous examinations of sinus puncture aspirates from patients with chronic sinusitis have yielded mixed findings, varying from the absence of anaerobes to anaerobes constituting 56% of all pathogens isolated [13, 14, 17–19]. When adequate sample proceedings and cultivation methods are used, anaerobes can be isolated in more than half of all cases [20]; the leading anaerobic strains were pigmented *Prevotella* spp., *Fusobacterium* spp. and GPAC. Aerobic and anaerobic *β*-lactamase–producing bacteria (BLPB), such as *S. aureus, Haemophilus, Prevotella, Porphyromonas* and *Fusobacterium* spp. were isolated from more than one-third of patients in different investigations [21–25]. Brook established the microbiological characteristics of acute exacerbation of chronic sinusitis (AECS) in an Academic Medical Center compared with chronic sinusitis [24]. He reported 32 patients with chronic sinusitis and 30 patients with AECS and found a total of 81 various isolates (33 aerobic and 48 anaerobic), which were recovered from the 32 cases (2.5 per specimen) with patients of chronic sinusitis. Aerobes alone were recovered in 8 specimens (25%), anaerobes only were isolated in 11 cases (34%), and mixed aerobes and anaerobes were recovered in 13 samples (41%). The predominant aerobic and facultative bacteria were members of Enterobacterales and *S. aureus*, while predominant anaerobic bacteria were GPAC, *Fusobacterium* spp., anaerobic Gram-negative bacilli and *Cutibacterium acnes* [24]. In a study by Erkan *et al.*, a total of 89 isolates (40 aerobic and facultative anaerobes, and 49 anaerobes) were recovered from the 30 patients (3.0 per specimen) with AECS: aerobes were recovered in 8 instances (27%), anaerobes only in 11 (37%) and mixed aerobes and anaerobes were recovered in 11 cases (37%). The predominant aerobes in his study were *S. pneumoniae*, Enterobacterales and *S. aureus.* This investigation demonstrates that the organisms isolated from patients with AECS were predominantly anaerobic and were similar to those generally recovered in patients with CRS [25]. However, aerobic bacteria that are usually found in acute infections (e.g. *S. pneumoniae, H. influenzae* and *M. catarrhalis)* can also emerge in some of the episodes of AECS [25]. In contrast to these studies, Bhattacharyya *et al.* found that both anaerobes and aerobic species could be recovered from both diseased and the non-diseased contralateral side of patients with chronic rhinosinusitis, casting doubt on the aetiological role of bacteria in CRS; their main finding was that anaerobes are more prevalent in infections secondary to dental problems [26]. Jun Kim *et al.* investigated the bacteriology and antimicrobial susceptibility of maxillary sinus aspirates from 81 patients [27]. Aerobes were isolated from 58.0% of the cultures from the middle meatus and from 48.1% of those from the maxillary sinus: *S. aureus, H. influenzae* and *S. pneumoniae* were the most prevalent aerobic pathogens. Anaerobes were only isolated from 8.6% of the cultures from the middle meatus and from 18.5% of the cultures from the maxillary sinus. In this investigation the predominant anaerobic organisms were *Prevotella* spp. and GPAC in adults, but interesting, none of these isolates were cultured in children. A high rate of concordance of the middle meatus and maxillary sinus was noted and monomicrobial infection was most commonly observed [27]. An open-label, multicenter study was performed by Finegold *et al.* in 2002 to assess culturable bacteriologic findings associated with chronic bacterial maxillary sinusitis in adults [28]. Seventy aerobic (52.2%) and 64 anaerobic (47.8%) pathogens were recovered from clinically evaluable patients at baseline (before therapy). The most commonly isolated anaerobic bacteria were *Prevotella* spp. (31.1%), GPAC (21.9%) and *Fusobacterium* spp. (15.6%), their findings consistent with results of other earlier studies. The aerobes most frequently recovered included *Streptococcus* spp. (21.4%), *H. influenzae* (15.7%), *Pseudomonas aeruginosa* (15.7%), *S. aureus* and *M. catarrhalis* (10.0% each). Recurrences for signs or symptoms of bacterial maxillary sinusitis associated with anaerobes were twice as frequent as were those associated with aerobes when counts of anaerobes were above or equal to 10^3^ CFU/mL [28]. In addition, a pathogenic role for *Granulicatella* spp. in chronic sinusitis cases was documented for the first time in this study. Brook and Frazier correlated the microbiological findings with the history of sinus surgery in 108 patients with chronic maxillary sinusitis and found a higher rate of isolation of *P. aeruginosa* and other Gram-negative bacilli in patients with previous sinus surgery [29, 30]. Anaerobes were, however, isolated significantly more frequently in patients who did not have prior surgery. Brook evaluated the microbiology of 13 chronically infected frontal [30], seven sphenoid [31] and 17 ethmoid sinuses [32]: anaerobic bacteria were recovered in more than two-thirds of the patients. In these studies, the predominant anaerobic species included *Prevotella*, GPAC and *Fusobacterium* spp., the main aerobic organisms were Gram-negative bacilli (H. *influenzae, K. pneumoniae, E. coli* and *P. aeruginosa)* [30–32]*.* Nadel *et al.* isolated Gram-negative enteric rods more commonly in patient with a history of previous surgery or those who had sinus irrigation, *P. aeruginosa* was also more frequent in patients who received systemic steroids [21]. Other studies have also noted this shift toward Gram-negative aerobic organisms in patients who had been extensively and repeatedly treated [27, 28, 33]. According to the recent study of Little *et al.* the microbiology of odontogenic sinusitis was distinctly different from cases of non-odontogenic sinusitis: odontogenic-issue sinus infections are generally polymicrobial with obligate anaerobic bacteria predominantly present in cultures, commonly including *GPAC*, Prevotella and Fusobacterium spp. [34]. These higher rates of mixed aerobic and anaerobic infections among patients with odontogenic sinusitis have been well documented in the literature [35, 36]. Zirk *et al.* reviewed 121 cases of odontogenic sinusitis and noted that 70% demonstrated anaerobic isolates and 30% aerobes or facultative anaerobes [37]. The variable growth of microbes in samples may also be due to prior exposure of various broad-spectrum antibiotics in patients involved in the studies.

The role of anaerobic bacteria in chronic sinusitis is supported by their ability to induce chronic sinusitis in a rabbit by intra-sinus inoculation of *B. fragilis* and the rapid production of serum immunoglobulin G (IgG) antibodies against this organism in the infected animals. In a recent investigation of Jyonouchi *et al.*, the study group induced chronic sinusitis successfully in animal models via intra-sinus inoculation of a *B. fragilis strain* [38]. These authors subsequently identified IgG antibodies against the inoculated *B. fragilis* in the infected rabbits. In addition the other study, the immune response, specific IgG antibodies to 2 anaerobic bacteria *(F. nucleatum* and *Prevotella intermedia)* in patients with chronic maxillary sinusitis have been observed [39], so these findings further support a pathogenic role for anaerobes in chronic sinusitis. Antibody levels to these organisms declined in the individuals who responded to therapy and were cured, but did not decline in those who failed treatment. In the studies which used appropriate anaerobic cultivation methods and laboratory techniques for identification, the anaerobic bacteria accounted for 25–56% of the isolates. A recent study using sequencing the species-specific 16S ribosomal DNA fragment for genetic identification of bacteria illustrated the recovery of anaerobes in half of the 18 patients with chronic sinusitis [40].

In our previous study, performed among children after adenoidectomy, the cultivable bacterial composition from nasopharyngeal swabs and from the removed adenoid tissue in the same patient group were compared [41]. The viable bacterial cells (number of colony-forming units) were quantified and the composition of isolated bacteria from both types of samples was also determined in parallel. Our findings showed that the culture results of nasopharyngeal swabs and inner part of the adenoid tissue are in close correlation: polymicrobial aerobic-anaerobic flora was present in all cases. The predominant aerobic isolates in all two groups were the members of the ‘classical triad,’ namely *S. pneumoniae, H. influenzae* and *M. catarrhalis.* Most common anaerobic strains recovered from the adenoid tissues were *Peptostreptococcus* spp., *Prevotella* spp. and *Fusobacterium* spp. [41].

Our present study of adult CRS patients illustrates the importance of obtaining correct samples from patients with CRS for both aerobic and anaerobic cultures to guide the selection of the proper antimicrobial therapy and to prevent possible life threatening-sequelae. Microbiologic studies of chronic sinusitis often show that the infection is polymicrobial, with the isolation of 1–6 isolates per specimen [21–36]. In this study, the distribution of bacterial number was higher, this number was 1–10 (average: 2.6) and anaerobes made up 51.5% of the pathogens isolated. Black-pigmented species, including *Prevotella* and *Porphyromonas* spp., GPAC and *Fusobacterium* spp. accounted for 63% all of the anaerobic pathogens isolated, a finding consistent with the results of some of the data of the literature which noted a diversity of aerobic and anaerobic bacteria similar to that in our study. Our higher isolation rate of *Actinomyces* spp. could be attributed to the applied longer incubation period (6–8 days) [42]. The distribution of aerobic and/or facultative anaerobic pathogens in the present investigation was consistent with that seen in some of the other studies of chronic sinusitis [21–36]. Similar to the data available in the literature, *S. pneumoniae* (50.6%), *H. influenzae* (36.7%) and *M. catarrhalis* (7.6%) were among the most frequently isolated aerobic and/or facultative anaerobic pathogens. Isolation of Gram-negative enteric rods, including *P. aeruginosa, K. pneumoniae, Proteus mirabilis, Enterobacter* spp. and *E. coli* were also reported in some other studies [21]. Because these bacteria are rarely isolated from sinus cultures obtained from healthy individuals, their recovery from these symptomatic patients suggests their pathogenic role. These organisms may have been selected out following administration of antimicrobial therapy in patients with chronic sinusitis. Furthermore, consistent with the findings of other published studies, a wide variety of other aerobes and/or facultative anaerobic pathogens were also recovered *(P. aeruginosa*, members of Enterobacterales and fungi). The emergence of new pathogens in all instances, mostly strict anaerobes, generated a polymicrobial infection. This type of infection is one of synergistical nature, and may be more difficult to eradicate with narrow spectrum antimicrobial agents [15, 43]. In such mixed infection, mutual enhancement of bacterial growth, and ‘protection’ of penicillin-susceptible isolates by beta-lactamase produced by relevant bacteria, may contribute to the chronicity of the infection, and the difficulty in its eradication [15, 16].

## CONCLUSIONS

This is the first published account of the detailed microbiology of adult-chronic sinusitis in Hungary. The absence of accurate epidemiological data in Hungary on CRS contrasts with the more abundant information on microorganisms, diagnosis and treatment options for these conditions. Our understanding of microorganisms in the paranasal sinus is still incomplete, although there is some association between the viral, fungal and bacterial microorganisms and CRS, the exact nature and importance of the relationship is still unclear. The microbiology of sinusitis is influenced by the previous antimicrobial therapy, vaccinations, and the presence of the conventional commensal flora, capable of interfering with the growth of pathogens. The microbial flora of chronic sinusitis is affected by previous antibiotic administration, past vaccinations and the presence of normal flora that can suppress the emergence of pathogenic species. In some cases, the baseline chronic sinusitis worsens suddenly or causes new symptoms. This acute exacerbation of chronic sinusitis is often polymicrobial as well, with anaerobic bacteria predominating.

## Figures and Tables

**Table 1.** Distribution of *n =* 106 anaerobic bacterial strains recovered from patients with chronic bacterial sinusitis

Species	No. of isolates	% of all anaerobic strains
*Prevotella*		**20.8**
*P. intermedia*	6	
*P. loescheii*	5	
*P. denticola*	4	
*P. bivia*	2	
*P. melaninogenica*	3	
*P. buccae*	2	
*Porphyromonas*		**4.7**
*P. gingivalis*	4	
*P. assaccharolytica*	1	
*Fusobacterium*		**20.8**
*F. nucleatum*	12	
*F. necrophorum*	5	
*F. mortiferum*	2	
*Bacteroides*	3.8	
*B. ureolyticus*	2	
*B. fragilis*	2	
Others:		**10.4**
*Veillonella parvula*	2	
*Leptotrichia buccalis*	2	
*Eikenella corrodens*	2	
*Solobacterium mooreii*	5	
*Actinomyces*		**11.2**
*A. viscosus*	3	
*A odontolyticus*	5	
*A. meyeri*	3	
*A. naeslundii*	2	
*Cutibacterium*		**8.5**
*C. acnes*	8	
*C. propionicum*	1	
GPAC		**15.1**
*P. anaerobius*	6	
*P. micra*	6	
*F. magna*	4	
*Clostridium*		**2.8**
*C. sordellii*	1	
*C. butyricum*	2	
*Eggerthella lenta*	2	**1.9**

Percentageratios ofvarious bacterialgenera are shownin boldface(%).
